# Dynamic cytokine monitoring enhances CAP severity scores in elderly patients: a prospective pilot study

**DOI:** 10.1007/s11739-025-03975-7

**Published:** 2025-05-27

**Authors:** Cheng-Han Chen, Yi-Tzu Lee, Ching-Fen Shen, Chao-Min Cheng

**Affiliations:** 1 Department of Emergency Medicine, Taipei Veterans General Hospital, Taipei, Taiwan; 2 School of Medicine, National Yang Ming Chiao Tung University, Taipei, Taiwan; 3 Department of Pediatrics, National Cheng Kung University Hospital, College of Medicine, National Cheng Kung University, Tainan, Taiwan; 4 Institute of Clinical Medicine, College of Medicine, National Cheng Kung University, Tainan, Taiwan; 5 Institute of Biomedical Engineering, National Tsing Hua University, Hsinchu, Taiwan

**Keywords:** Community-acquired pneumonia, Cytokine dynamics, Elderly, Interleukin-6, Mortality prediction, Risk stratification

## Abstract

Current severity scoring systems (PSI and CURB-65) have limitations in risk stratification for elderly patients with community-acquired pneumonia (CAP). Given the complex immune responses in elderly populations, dynamic biomarker monitoring may provide additional prognostic value. This study evaluates whether integrating early cytokine dynamics with traditional severity scores improves mortality prediction in elderly CAP patients. This prospective observational study included 81 CAP patients aged ≥ 65 years. Multiple cytokines were measured at admission and within 48 h. Traditional severity scores (PSI and CURB-65) were calculated at baseline. Patients were categorized into survival (n = 67) and mortality (n = 14) groups. The predictive value of cytokine dynamics alone and in combination with severity scores was assessed using ROC curve analysis. Among measured cytokines, IL-6 demonstrated significant prognostic value. The mortality group showed an 88% increase in IL-6 levels within 48 h, contrasting with a 49% decrease in survivors (p = 0.040). While individual PSI (AUC = 0.6631) and CURB-65 (AUC = 0.6231) showed modest discrimination, integration with IL-6 dynamics significantly improved predictive accuracy (PSI + IL-6: AUC = 0.7676, p = 0.0017; CURB-65 + IL-6: AUC = 0.7564, p = 0.0027). Early dynamic monitoring of cytokines, particularly IL-6, significantly enhances the prognostic accuracy of traditional severity scores in elderly CAP patients. This pilot study suggests that this integrated approach provides a more precise risk stratification tool, potentially enabling more personalized clinical decision-making. Larger multicenter studies are warranted to validate these findings and establish standardized cutoff values for clinical implementation.

**Supplementary Information:**

The online version contains supplementary material available at 10.1007/s11739-025-03975-7.

## Introduction

Community-acquired pneumonia (CAP) remains a significant cause of morbidity and mortality in the elderly population. Due to age-related changes in immune function, often referred to as immunosenescence or"inflammaging,"older adults exhibit heightened susceptibility to infections [1, 2]. Furthermore, elderly patients with CAP frequently present with atypical symptoms, complicating accurate diagnosis and risk assessment [3]. The pathophysiology of CAP is intricate, involving complex interactions between host immune responses and pathogens [4].

Prognostic tools currently used for CAP, such as the Pneumonia Severity Index (PSI) and CURB-65, may have limitations in assessing risk among older patients. These scoring systems place substantial emphasis on age and comorbidities, potentially leading to inaccuracies in risk stratification for this demographic [5, 6]. This limitation underscores the need for additional prognostic markers to provide more nuanced information regarding disease severity and progression.

The pathophysiology of CAP in elderly patients is particularly complex, exhibiting distinct inflammatory response characteristics. Older individuals with pneumonia often display abnormal immune response patterns, characterized by elevated levels of certain pro-inflammatory cytokines but relatively blunted overall inflammatory responses, such as lower C-reactive protein (CRP) levels [1, 2, 7]. This attenuated systemic inflammatory response not only increases susceptibility to infections but also adversely impacts prognosis [7]. Therefore, a systematic understanding of these age-associated immune responses is essential for improving the management of bacterial pneumonia in older adults.

Cytokines, as key regulators of the immune system, play a pivotal role in the progression and prognosis of pneumonia. These include, but are not limited to, pro-inflammatory cytokines such as interleukin-1 beta (IL-1β), interleukin-6 (IL-6), interleukin-8 (IL-8), and tumor necrosis factor-alpha (TNF-α); anti-inflammatory cytokines such as interleukin-10 (IL-10); chemokines such as monocyte chemoattractant protein-1 (MCP-1) and macrophage inflammatory protein-1 alpha (MIP-1α); and growth factors such as granulocyte–macrophage colony-stimulating factor (GM-CSF) [8]. Previous studies have suggested that the expression patterns of these cytokines may reflect disease severity and prognosis [9].

Of particular interest, a recent meta-analysis of sepsis patients highlighted that dynamic changes in cytokine levels might better predict mortality than their absolute concentrations [10]. This finding suggests that monitoring cytokine dynamics could have greater clinical value than single-time-point measurements in evaluating disease progression and prognosis [11, 12]. However, it remains unclear which specific cytokines and dynamic patterns are most predictive in elderly patients with CAP. This gap in knowledge emphasizes the importance of systematically evaluating dynamic changes in multiple cytokines to identify biomarkers most suitable for prognostic assessment in elderly CAP patients.

The precise relationship between cytokine dynamics and clinical outcomes in elderly CAP patients remains a critical knowledge gap. While prior research has explored cytokine levels in CAP patients, most studies have focused on single-time-point measurements or static analyses, often in younger or mixed-age populations [8, 9].

This pilot study aims to investigate the potential prognostic value of dynamic changes in multiple inflammatory mediators in elderly CAP patients through comprehensive cytokine array analysis. By examining temporal variations in a broad spectrum of cytokines and integrating these findings with existing severity scoring systems, we seek to identify novel biomarker patterns that may enhance risk stratification. Through this exploratory approach, we aim to establish a more accurate prognostic framework that combines dynamic cytokine monitoring with current clinical scoring systems, potentially enabling more precise risk assessment and personalized therapeutic strategies for this vulnerable population.

## Methods

### Study design and participants

This prospective, observational study was conducted from July 2022 to June 2023 in the Emergency Department of Taipei Veterans General Hospital. The study protocol was approved by the Institutional Review Board (IRB approval numbers: 2021-06-014 AC and 2023-01-023BCF) and was conducted in accordance with the principles outlined in the Declaration of Helsinki. Written informed consent was obtained from all participants prior to enrollment.

The primary inclusion criteria were as follows: (1) age of 65 years or older; (2) presentation with community-acquired pneumonia (CAP) at the emergency department, excluding those with confirmed COVID-19 infection; (3) diagnosis of pneumonia based on the presence of at least two respiratory symptoms or signs (e.g., cough, fever, hypothermia, purulent sputum, or respiratory secretions) and radiographic confirmation of infiltrates on chest X-ray as interpreted by attending physicians [13]; and (4) CAP diagnosis in accordance with the standards of the Infectious Diseases Society of America (IDSA) [14]. Exclusion criteria included: (1) imaging findings suggestive of lung cancer or pulmonary metastases; (2) active cancer patients undergoing chemotherapy or targeted therapy; (3) immunocompromised status, defined as a CD4 count < 200 cells/μL, long-term steroid use exceeding 20 mg/day, or ongoing immunosuppressive therapy; (4) concurrent pancreatitis; (5) transfer from another hospital with symptoms persisting for more than five days; (6) non-compliance with the study protocol; (7) unwillingness to participate; or (8) inability to provide informed consent; (9) death prior to the second cytokine measurement.

Standard diagnostic and treatment procedures for CAP were applied to all participants. The treatment teams were blinded to cytokine test results and operated independently of the research team. At baseline, complete blood counts and biochemical tests were obtained upon admission to the emergency department, and standard chest X-rays and/or computed tomography (CT) scans were performed. The severity of pneumonia was evaluated using the Pneumonia Severity Index (PSI) and CURB-65 scores. An initial serum cytokine measurement was conducted at admission, followed by a second cytokine measurement within 48 h.

For analysis, patients were stratified into two groups: those who died during hospitalization and those who survived and were discharged. The dynamic changes in cytokine levels were compared between these groups. Secondary outcomes, including the incidence of respiratory failure, length of intensive care unit stay, and total hospital stay duration, were also evaluated.

### Cytokine analysis

Blood samples were collected at two predefined time points: at admission to the emergency department (baseline) and within 48 h after admission (follow-up). Serum samples were processed by centrifugation and stored at − 80 °C until further analysis. Cytokine measurements were performed using the Bio-Plex Pro Human Cytokine 27-plex Assay (Bio-Rad Laboratories, Hercules, CA, #M500KCAF0Y). The analysis included the following cytokine groups:

- Pro-inflammatory cytokines: interleukin-1 beta (IL-1β), interleukin-2 (IL-2), interleukin-6 (IL-6), interleukin-8 (IL-8), interleukin-12p70 (IL-12p70), and interleukin-17a (IL-17a)
- Anti-inflammatory cytokines: interleukin-10 (IL-10) and interleukin-13 (IL-13)
- Growth factors: granulocyte–macrophage colony-stimulating factor (GM-CSF)
- Other inflammatory mediators: tumor necrosis factor-alpha (TNF-α), interferon-gamma (IFN-γ), interferon gamma-induced protein 10 (IP-10), monocyte chemoattractant protein-1 (MCP-1), and macrophage inflammatory protein-1 alpha (MIP-1α)

### Dynamic change calculation

Dynamic changes in cytokine levels were calculated as relative changes from baseline using the formula:

$$Dynamic\,Change\,Ratio = \frac{{{\text{Follow - up Value}} - {\text{Baseline Value}}}}{{{\text{Baseline Value}}}}$$

This standardized methodology allows for direct comparisons across cytokines while accounting for inter-patient variability in baseline cytokine levels. A positive ratio indicates an increase in cytokine levels from baseline, whereas a negative ratio reflects a decrease. By normalizing the data, this approach provides a robust framework for evaluating cytokine dynamics across the study cohort.

### Statistical analysis

All statistical analyses were conducted using IBM SPSS (version 29.0, IBM Corporation, USA) and GraphPad Prism (version 10.2.3, GraphPad Software Inc., San Diego, California, USA). Statistical significance was defined as a two-sided p-value of less than 0.05. Continuous variables were summarized as mean ± standard deviation or medians with interquartile ranges, while categorical variables were expressed as frequencies and percentages. Comparisons of non-normally distributed variables across groups were performed using the Mann–Whitney U test or the Kruskal–Wallis H test. Dynamic changes in cytokine levels were calculated as relative changes from baseline, defined as ((follow-up value – baseline value)/baseline value), and compared between the survival and mortality groups. Receiver Operating Characteristic curves were constructed to assess the predictive performance of individual parameters, including cytokine dynamic changes, Pneumonia Severity Index, and CURB-65 scores; combined models integrating cytokine dynamics with Pneumonia Severity Index or CURB-65 scores; and the incremental value of incorporating cytokine dynamics into existing severity scoring systems. The area under the curve values were computed to evaluate discriminatory capacity, with 95% confidence intervals provided.

## Results

### Patient characteristics and clinical outcomes

This study included 81 elderly patients diagnosed with community-acquired pneumonia, of whom 67 (82.7%) survived and 14 (17.3%) did not. As shown in Table 1, baseline demographic characteristics showed a higher proportion of males (78.6% vs. 68.7%) and slightly older age (86.00 ± 7.78 years vs. 82.21 ± 14.38 years) in the non-survivor group compared to survivors, consistent with known risk factors for poor outcomes in pneumonia.

**Table 1:** Patient characteristics and clinical outcomes

Variable	Survival	Mortality	p value
Total	67	14	
*Demographic characteristics*			
Male, n (%)	46 (68.7)	11 (78.6)	0.667
Age, median [IQR]	85.00 [76.50, 92.00]	85.50 [78.50, 93.25]	0.516
BMI, median [IQR]	21.00 [18.64, 23.37]	17.25 [15.55, 21.20]	0.035*
Nursing facility, n (%)	15 (22.4)	2 (14.3)	0.723
*Medical history and comorbidities*			
CCI score, median [IQR]	6.00 [4.00, 7.00]	6.00 [5.00, 7.00]	0.314
COPD (%)	10 (15.2)	4 (28.6)	0.254
Asthma (%)	4 (6.0)	1 (7.1)	1.000
Ex-smoker (%)	12 (17.9)	3 (21.4)	1.000
Diabetes mellitus (%)	18 (26.9)	5 (35.7)	0.732
Liver disease (%)	4 (6.0)	2 (14.3)	0.603
CVA (%)	7 (10.6)	2 (14.3)	0.653
CHF (%)	11 (16.7)	2 (14.3)	1.000
CAD (%)	2 (3.0)	2 (14.3)	0.139
CKD (%)	4 (6.0)	1 (7.1)	1.000
*Disease severity scores*			
PSI, median [IQR]	137.00 [102.50, 172.00]	151.50 [139.75, 192.75]	0.062
CURB-65, median [IQR]	2.00 [2.00, 3.00]	3.00 [2.00, 4.00]	0.103
*Clinical outcomes*			
LOS (days), median [IQR]	15.00 [9.00, 24.50]	5.00 [4.00, 16.25]	0.038*
Length of CU Stay, median [IQR]	14.50 [8.00, 25.00]	4.50 [3.25, 16.00]	0.032*
ICU admission (%)	28 (41.8)	10 (71.4)	0.084
Ventilation support (%)^1^	17 (27.4)	7 (53.8)	0.126

*SD* standard deviation; *BMI* body mass index; *CCI* Charlson comorbidity index; *IQR* interquartile range; *COPD* chronic obstructive pulmonary disease; *CVA* cerebrovascular accident; *CHF* congestive heart failure; *CAD* coronary artery disease; *CKD* chronic kidney disease; *PSI* pneumonia severity index; *CURB-65* confusion, urea, respiratory rate, blood pressure, age ≥ 65; *LOS* length of stay; *ICU* intensive care unit

^1^Ventilation support: defined as the need for mechanical ventilation or non-invasive respiratory support such as bilevel positive airway pressure or high flow nasal cannula

Bold values indicate statistical significance (*p* 0.05)

### Comorbidities and disease severity

The burden of comorbidities, as assessed by the Charlson Comorbidity Index, showed a median score of 6.00 in both groups. However, non-survivors had higher prevalence of several important comorbidities, including chronic obstructive pulmonary disease (28.6% vs. 15.2%), coronary artery disease (14.3% vs. 3.0%), and diabetes mellitus (35.7% vs. 26.9%). These patterns align with the clinical understanding that certain comorbidities contribute to poorer outcomes in pneumonia patients (Table 1).

Disease severity scores were notably higher in the non-survivor group, with median Pneumonia Severity Index of 151.50 compared to 137.00 in survivors, and median CURB-65 score of 3.00 versus 2.00. These elevated severity scores reflect the greater disease burden at presentation in patients who ultimately did not survive.

### Clinical outcomes

Clear differences were observed in the hospital course metrics between groups. The median length of hospital stay was substantially shorter in the non-survivor group compared with survivors (5.00 days vs. 15.00 days; p = 0.038), as was the duration of critical care unit stay (4.50 days vs. 14.50 days; p = 0.032), reflecting the rapid clinical deterioration leading to earlier mortality in these patients.

Non-survivors demonstrated a more severe clinical course, with notably higher intensive care unit admission rates (71.4% vs. 41.8%) and greater need for ventilatory support (53.8% vs. 27.4%), reflecting the overall greater disease burden in this group (Table 1). 

### Baseline clinical, biochemical, and cytokine profiles

Table 2 summarizes the baseline characteristics of elderly patients with community-acquired pneumonia at the time of emergency department presentation, comparing traditional severity markers with a comprehensive cytokine profile between survival and mortality groups. Among conventional clinical parameters, respiratory rate was notably elevated in the mortality group (28.00 [26.00–30.00] vs. 24.00 [20.00–30.00] breaths per minute; p = 0.017), reflecting greater respiratory distress. Other vital signs showed clinically relevant patterns, including relatively higher heart rate (107.00 vs. 102.00 beats/min) and lower mean arterial pressure (88.82 vs. 95.17 mmHg) in non-survivors, consistent with the physiological stress response typically observed in more severely ill patients.

**Table 2:** Baseline clinical, biochemical, and cytokine profiles

Variable	Survival, median [IQR]	Mortality, median [IQR]	p value
Vital signs			
Temp, °C	37.30 [36.40, 38.10]	37.10 [36.42, 37.98]	0.793
RR, breaths/min	24.00 [20.00, 30.00]	28.00 [26.00, 30.00]	0.017*
HR, beats/min	102.00 [87.00, 115.50]	107.00 [91.25, 125.25]	0.666
SBP, mmHg	135.00 [117.00, 153.00]	135.50 [106.75, 144.50]	0.339
MAP, mmHg	95.17 [82.83, 105.08]	88.82 [70.75, 95.50]	0.121
SpO_2_/FiO_2_ ratio	4.33 [3.06, 4.52]	4.10 [2.74, 4.33]	0.237
Traditional inflammatory markers			
WBC, × 10⁹/L	10.48 [8.40, 14.495]	12.565 [9.79, 20.275]	0.309
CRP, mg/dL	8.71 [3.83, 15.06]	11.10 [5.31, 14.80]	0.582
PCT, ng/mL^1^	0.20 [0.12, 0.49]	0.66 [0.36, 1.82]	0.126
Baseline cytokine profiles (pg/mL)			
*Pro-inflammatory cytokines*			
Interleukin-1β	1.48 [0.32, 4.15]	0.45 [0.30, 1.10]	0.290
Interleukin-2	4.00 [1.20, 8.21]	1.92 [0.96, 4.27]	0.520
Interleukin-6	79.93 [31.81, 242.16]	53.25 [16.63, 251.21]	0.545
Interleukin-8	39.37 [21.00, 84.55]	56.30 [31.23, 213.92]	0.093
Interleukin-12 (p70)	3.58 [3.58, 11.92]	3.58 [1.35, 4.33]	0.188
Interleukin-17a	6.83 [4.72, 10.35]	5.00 [2.56, 7.23]	0.334
*Anti-inflammatory cytokines*			
Interleukin-10	15.02 [6.36, 43.68]	4.64 [2.65, 90.32]	0.115
Interleukin-13	0.17 [0.17, 1.37]	0.17 [0.17, 0.94]	0.644
*Other inflammatory mediators*			
GM-CSF	2.03 [0.20, 5.26]	1.52 [0.20, 5.45]	0.683
TNF-α	68.48 [35.85, 110.66]	48.06 [25.43, 126.04]	0.587
IFN-γ	32.95 [11.75, 107.84]	24.25 [9.85, 70.85]	0.680
IP-10	644.48 [260.79, 1365.14]	898.87 [500.32, 1581.18]	0.249
MCP-1	162.91 [63.94, 421.60]	158.48 [92.78, 503.23]	0.760
MIP-1α	5.40 [3.42, 8.36]	6.82 [4.47, 17.36]	0.147

*SD* standard deviation; *Temp* temperature; °*C* degrees celsius; *RR* respiratory rate; *HR* heart rate; *SBP* systolic blood pressure; *MAP* mean arterial pressure; *mmHg* millimeters of mercury; *SpO*_*2*_ oxygen saturation; *FiO*_*2*_ fraction of inspired oxygen; *ED* emergency department; *WBC* white blood cell count; *CRP* C-reactive protein; *GM-CSF* granulocyte–macrophage colony-stimulating factor; *TNF-α* tumor necrosis factor alpha; *IFN-γ* interferon gamma; *MCP-1* monocyte chemoattractant protein-1; *MIP-1α* macrophage inflammatory protein-1 alpha; *pg/mL* picograms per milliliter; *ng/mL* nanograms per milliliter; *mg/dL* milligrams per deciliter; × *10⁹/L* billion per liter

^1^PCT data were available for only 46 patients (39 survivors and 7 non-survivors)

Bold values indicate statistical significance (*p* 0.05)

At baseline, traditional inflammatory markers showed patterns consistent with more pronounced inflammation in the mortality group, with elevated white blood cell counts (12.565 [9.79–20.275] vs. 10.48 [8.40–14.495] × 10^9^/L) and C-reactive protein levels (11.10 [5.31–14.80] vs. 8.71 [3.83–15.06] mg/dL). In the subset of patients with procalcitonin measurements available (n = 46), baseline levels were also higher in the mortality group (0.66 [IQR: 1.47] vs. 0.2 [IQR: 0.37] ng/mL, p = 0.126). These differences in inflammatory response underscored the importance of examining dynamic changes rather than single time-point measurements in elderly CAP patients.

Baseline cytokine analysis revealed differential inflammatory patterns between groups. IL-8 levels were considerably higher in the mortality group (56.30 [31.23–213.92] vs. 39.37 [21.00–84.55] pg/mL), consistent with its role in neutrophil recruitment during severe infections. Similarly, IL-6 baseline levels showed variability between groups (mortality: 53.25 [16.63, 251.21] vs. survival: 79.93 [31.81, 242.16] pg/mL), highlighting the heterogeneity of inflammatory responses in elderly patients and the limitations of single time-point cytokine measurements for prognostication.

### Dynamic changes in cytokines and inflammatory markers

Table 3 summarizes the dynamic changes in inflammatory markers and cytokine profiles observed between the initial presentation and follow-up assessments. The mean time interval for follow-up measurements was comparable between the mortality and survival groups (15.46 [9.88, 17.40] hours vs. 14.78 [11.37, 20.69] hours, p = 0.520), ensuring valid temporal comparisons.

**Table 3:** Dynamic changes in cytokines and inflammatory markers

Variable	Survival, median [IQR]	Mortality, median [IQR]	p value
Time interval between tests (hours)			
Time interval	14.78 [11.37, 20.69]	15.46 [9.88, 17.40]	0.520
Changes in traditional inflammatory markers (ratio change)			
WBC, × 10⁹/L	– 0.18 [– 0.30, 0.00]	– 0.04 [– 0.26, 0.16]	0.446
CRP, mg/dL	0.10 [– 0.04, 0.44]	0.24 [– 0.04, 0.71]	0.606
PCT, ng/mL^1^	0.14 [– 0.16, 0.78]	0.38 [– 0.30, 1.48]	0.939
Changes in cytokine levels (ratio change)			
*Pro-inflammatory cytokines*			
Interleukin-1β	0.00 [– 0.30, 0.30]	0.53 [– 0.17, 1.07]	0.121
Interleukin-2	0.00 [– 0.36, 0.50]	0.83 [– 0.35, 1.41]	0.299
Interleukin-6	– 0.49 [– 0.76, – 0.10]	0.88 [– 0.35, 1.49]	0.040*
Interleukin-8	– 0.02 [– 0.41, 0.25]	0.32 [– 0.30, 1.34]	0.183
Interleukin-12 (p70)	0.00 [– 0.07, 0.96]	0.00 [– 0.27, 1.21]	0.985
Interleukin-17a	– 0.15 [– 0.38, 0.31]	0.30 [– 0.05, 0.49]	0.214
*Anti-inflammatory cytokines*			
Interleukin-10	– 0.25 [– 0.57, 0.11]	0.28 [– 0.44, 1.07]	0.095
Interleukin-13	0.00 [0.00, 0.00]	0.03 [– 0.32, 2.02]	0.460
*Other inflammatory mediators*			
GM-CSF	0.00 [– 0.42, 0.61]	0.06 [– 0.33, 0.77]	0.581
TNF-α	0.09 [– 0.17, 0.31]	0.14 [– 0.15, 0.74]	0.596
IFN-γ	– 0.05 [– 0.50, 0.21]	0.09 [– 0.47, 0.21]	0.488
IP-10	0.08 [– 0.23, 0.40]	– 0.05 [– 0.35, 0.23]	0.366
MCP-1	– 0.06 [– 0.48, 0.31]	0.00 [– 0.58, 1.07]	0.760
MIP-1α	0.01 [– 0.19, 0.29]	0.12 [– 0.43, 0.93]	0.808

*SD* standard deviation; *ED* emergency department; *WBC* white blood cell count; *CRP* C-reactive protein; *GM-CSF* granulocyte–macrophage colony-stimulating factor; *TNF-α* tumor necrosis factor alpha; *IFN-γ* interferon gamma; *MCP-1* monocyte chemoattractant protein-1; *MIP-1α* macrophage inflammatory protein-1 alpha; *pg/mL* picograms per milliliter; *ng/mL* nanograms per milliliter; *mg/dL* Milligrams per deciliter*; × 10⁹/L* billion per liter

^1^PCT data were available for only 46 patients (39 survivors and 7 non-survivors)

Bold values indicate statistical significance (*p* 0.05)

Among all measured inflammatory parameters, IL-6 was the only marker demonstrating significant dynamic changes predictive of mortality. In the mortality group, IL-6 levels markedly increased (ratio change: 0.88 [− 0.35, 1.49]), contrasting with a decrease observed in the survival group (ratio change: − 0.49 [− 0.76, − 0.10], p = 0.040). This distinct pattern was not seen in traditional inflammatory markers. C-reactive protein levels (ratio change: 0.24 [− 0.04, 0.71] vs. 0.10 [− 0.04, 0.44], p = 0.606) and white blood cell counts (ratio change: − 0.04 [− 0.26, 0.16] vs. − 0.18 [− 0.30, 0.00], p = 0.446) did not show significant discriminatory changes. PCT levels exhibited numerical differences between survivors (0.14 [− 0.16, 0.78]) and non-survivors (0.38 [− 0.30, 1.48]); however, the limited availability of data precludes definitive conclusions.

Other cytokines, such as IL-8 (ratio change: 0.32 vs. − 0.02) and IL-10 (ratio change: 0.28 vs. − 0.25), tended to be numerically elevated among non-survivors compared to survivors, although these differences did not reach statistical significance. IL-8 levels tended to rise in non-survivors (ratio change: 0.32 [− 0.30, 1.34] vs. − 0.02 [− 0.41, 0.25], p = 0.183), and IL-10 levels exhibited an upward trend (ratio change: 0.28 [− 0.44, 1.07] vs. − 0.25 [− 0.57, 0.11], p = 0.095). Changes in additional inflammatory mediators, including GM-CSF, TNF-α, IFN-γ, and other interleukins, remained comparable between the two groups.

These findings underscored the potential of dynamic IL-6 changes within the first 48 h as a sensitive indicator of disease progression in elderly patients with community-acquired pneumonia, surpassing the predictive utility of traditional inflammatory markers or other cytokines.

### Prognostic value of cytokine dynamics in elderly CAP patients

To assess whether cytokine dynamics enhance the prognostic accuracy of traditional severity scores in elderly patients with community-acquired pneumonia, we performed both individual and combined analyses of risk factors.

In the individual analysis (Table 4, Section A), IL-6 dynamics demonstrated significant prognostic value. Patients with increased IL-6 levels exhibited a markedly higher mortality rate (36.00%) compared to those with decreased levels (8.93%), yielding an odds ratio of 5.39 (95% CI 1.69–18.83, p = 0.004).

**Table 4:** Integration of IL-6 changes with clinical severity scores for mortality risk assessment

Section A. Individual analysis of risk factors						
Score system	Category	N	Mortality, n	mortality rate, %	OR (95% CI)	P-value
IL-6 change^†^	Decreased	56	5	8.93%	1.0 (ref)	–
	Increased	25	9	36.00%	5.39 (1.69–18.83)	0.004*
PSI score^‡^	Low-moderate risk (< 90)	14	0	0.00%	1.0 (ref)	–
	High risk (90–129)	27	4	14.81%	5.55 (0.53–756.41)	0.175
	Very high risk (≥ 130)	40	10	25.00%	9.98 (1.14–1316.56)	0.035*
CURB-65 score^§^	Low risk (0–1)	16	0	0.00%	1.0 (ref)	–
	Moderate risk (2)	33	7	21.21%	9.34 (1.02–1241.92)	0.048*
	High risk (≥ 3)	32	7	21.88%	9.71 (1.06–1291.11)	0.043*

Section B. Combined analysis of PSI score and IL-6 change on mortality risk						
PSI risk (score)	IL-6 change	N	Mortality, n	mortality rate, %	OR (95% CI)	P-value
Low-moderate risk (< 90)	Decreased	12	0	0.00%	1.0 (ref)	–
	Increased	2	0	0.00%	5.00 (0.02–1045.22)	0.463
High risk (90–129)	Decreased	19	2	10.53%	3.57 (0.26–509.55)	0.372
	Increased	8	2	25.00%	9.62 (0.65–1406.34)	0.103
Very high risk (≥ 130)	Decreased	25	3	12.00%	3.89 (0.34–539.16)	0.315
	Increased	15	7	46.67%	22.06 (2.18–3007.59)	0.005*

Section C. Combined analysis of CURB-65 score and IL-6 change on mortality risk						
CURB-65 (score)	IL-6 change	N	Mortality, n	mortality rate, %	OR (95% CI)	P-value
Low risk (0–1)	Decreased	14	0	0.00%	1.0 (ref)	–
	Increased	2	0	0.00%	5.80 (0.03–1209.23)	0.425
Moderate risk (2)	Decreased	21	2	9.52%	3.72 (0.27–528.26)	0.353
	Increased	12	5	41.67%	21.27 (1.98–2936.97)	0.008*
High risk (≥ 3)	Decreased	21	3	14.29%	5.49 (0.47–760.46)	0.194
	Increased	11	4	36.36%	17.40 (1.54–2425.65)	0.018*

*IL-6* interleukin-6; *PSI* Pneumonia Severity Index; *CURB-65* confusion, urea nitrogen, respiratory rate, blood pressure, and age ≥ 65 years; *OR* odds ratio; *CI* confidence interval; *ref* reference

*Statistically significant at P < 0.05

^†^IL-6 change was defined as the difference between baseline and follow-up measurements

^‡^PSI score (Pneumonia Severity Index): calculated based on 20 clinical parameters including demographic factors, comorbid illnesses, physical examination findings, and laboratory/radiographic findings. Class I–III (< 90), Class IV (90–129), and Class V (≥ 130)

^§^CURB-65 score: one point each for confusion, urea > 7 mmol/L, respiratory rate ≥ 30/min, blood pressure (systolic < 90 mmHg or diastolic ≤ 60 mmHg), and age ≥ 65 years. Low risk (0–1), moderate risk (2), and high risk (≥ 3)

Bold values indicate statistical significance (*p* 0.05)

The combined analysis (Table 4, Sections B and C) indicated that integrating IL-6 dynamics with conventional scoring systems significantly improved risk stratification. Among patients with very high Pneumonia Severity Index (PSI) scores (≥ 130), those with increased IL-6 levels had a significantly higher mortality rate (46.67%) compared to those with decreased levels (12.00%), corresponding to an odds ratio of 22.06 (95% CI 2.18–3007.59, p = 0.005). Similarly, in the moderate-risk CURB-65 group (score 2), increased IL-6 levels were associated with significantly higher mortality (41.67%) compared to decreased levels (9.52%), with an odds ratio of 21.27 (95% CI 1.98–2936.97, p = 0.008). 

**Fig. 1:**
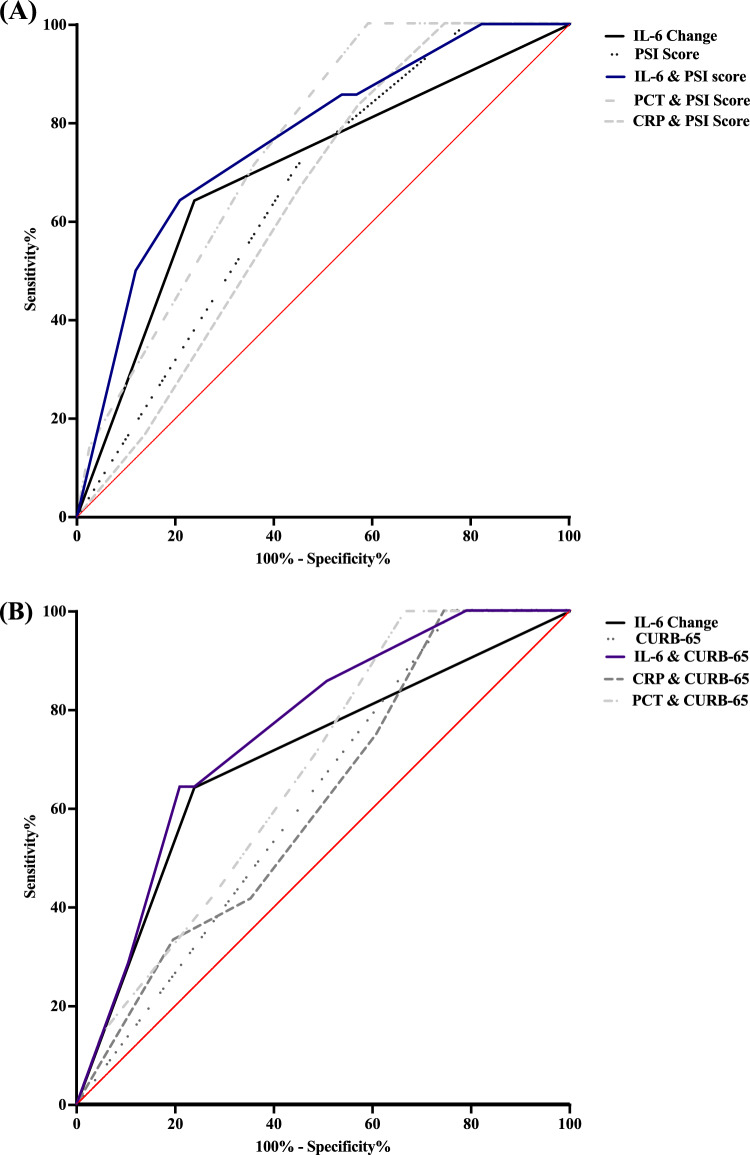
Receiver operating characteristic (ROC) curves for mortality prediction using PSI, CURB-65, and dynamic IL-6 changes **A** ROC curves comparing the prognostic performance of PSI alone (AUC = 0.6631, p = 0.056), IL-6 dynamics alone (AUC = 0.7020, p = 0.0179), and combinations of PSI with different inflammatory marker dynamics: PSI + IL-6 (AUC = 0.7676, p = 0.0017), PSI + PCT (AUC = 0.7527, p = 0.0348, n = 46), and PSI + CRP (AUC = 0.6471, p = 0.1152). The integration of IL-6 dynamics with PSI demonstrated strong discriminatory power in the full cohort. **B** ROC curves comparing the prognostic performance of CURB-65 alone (AUC = 0.6231, p = 0.1491), IL-6 dynamics alone (AUC = 0.7020, p = 0.0179), and combinations of CURB-65 with different inflammatory marker dynamics: CURB-65 + IL-6 (AUC = 0.7564, p = 0.0027), CURB-65 + PCT (AUC = 0.6740, p = 0.1463, n = 46), and CURB-65 + CRP (AUC = 0.6152, p = 0.2172). Among all combinations, CURB-65 + IL-6 demonstrated substantial improvement in mortality prediction (AUC = 0.7564, p = 0.0027). Note that PCT data were only available for a subset of patients (n = 46). Detailed statistical analyses are provided in Table 5 and Supplementary Table S4.

The comprehensive predictive model assessment using ROC curve analysis was presented in Table 5 and Fig. 1. The conventional severity scoring systems alone showed modest discriminatory power, with PSI achieving an AUC of 0.6631 (95% CI 0.5258–0.8004, p = 0.056) and CURB-65 reaching an AUC of 0.6231 (95% CI 0.4826–0.7637, p = 0.1491). Dynamic changes in IL-6 levels alone demonstrated improved predictive capability, with an AUC of 0.7020 (95% CI 0.5435–0.8606, p = 0.0179). When integrating IL-6 dynamics with traditional scoring systems, the predictive accuracy was further enhanced. The combination of PSI with IL-6 changes yielded the highest discriminatory power (AUC = 0.7676, 95% CI 0.6326–0.9026, p = 0.0017), followed by the combination of CURB-65 with IL-6 changes (AUC = 0.7564, 95% CI 0.6288–0.8840, p = 0.0027). These findings suggest that incorporating biomarker dynamics, particularly IL-6 changes, could potentially address some limitations of conventional severity scores in elderly CAP patients. To further contextualize the prognostic value of IL-6 dynamics, we evaluated models combining PSI and CURB-65 with CRP and PCT dynamics (Supplementary Table S4). These combinations yielded inferior or borderline AUC values, except for PSI plus PCT (AUC = 0.7527, p = 0.0348), which was limited by incomplete data (n = 46).

**Table 5:** Comparison of predictive models for mortality risk assessment

Model	AUC	SE	95% C.I (lower)	95% C.I (upper)	p value
PSI	0.6631	0.07005	0.5258	0.8004	0.056
CURB-65	0.6231	0.07173	0.4826	0.7637	0.1491
IL-6 change	0.7020	0.08091	0.5435	0.8606	0.0179^*^
CURB-65 & IL-6 Change	0.7564	0.06509	0.6288	0.8840	0.0027^**^
PSI & IL-6 change	0.7676	0.06889	0.6326	0.9026	0.0017^**^

*AUC* area under the curve; *SE* standard error; *CI* confidence interval; *PSI* Pneumonia Severity Index; *CURB-65* confusion, urea, respiratory rate, blood pressure, age ≥ 65 years; *IL-6* interleukin-6

*p < 0.05, **p < 0.01

The existing asterisk notation (*p 0.05, **p 0.01) is acceptable, as it clearly indicates statistical significance levels

## Discussion

This study aimed to evaluate the prognostic value of dynamic changes across a broad spectrum of cytokines in elderly patients with community-acquired pneumonia (CAP). Our findings identified IL-6 dynamics as the most significant predictor of mortality among all measured biomarkers. The mortality group exhibited a median IL-6 increase of 88% compared to a median decrease of 49% in the survival group (p = 0.040). Patients with increased IL-6 levels exhibited a substantially higher mortality rate (36.00%) compared to those with decreased levels (8.93%, OR = 5.39, p = 0.004). This strong association highlighted IL-6's rapid response characteristics which may allow for earlier identification of high-risk patients, potentially providing a critical window for therapeutic intervention before clinical decline becomes evident through other parameters.

### Comprehensive cytokine analysis and IL-6 dynamics

Through the analysis of multiple cytokines, including pro-inflammatory mediators (e.g., IL-1β, IL-8, TNF-α), anti-inflammatory mediators (e.g., IL-10), and chemotactic factors (e.g., MCP-1), IL-6 emerged as the sole cytokine with significant predictive value. In contrast, baseline levels and dynamic changes of other cytokines failed to differentiate survivors from non-survivors. These findings highlight IL-6's unique role in capturing the inflammatory trajectory in elderly CAP patients and its potential superiority as a prognostic biomarker.

IL-6 dynamics offer insights into disease progression that static measurements cannot provide. Elevated baseline IL-6 levels, often seen in elderly individuals due to immunosenescence and chronic inflammation, may obscure differences in static measurements [15–18]. Conversely, dynamic monitoring effectively accounts for temporal changes in IL-6 levels, reflecting acute inflammatory responses and their clinical implications [9, 19–21].

### Limitations of conventional scoring systems

Traditional severity scoring systems, such as the Pneumonia Severity Index (PSI) and CURB-65, demonstrated limited utility in our cohort of elderly patients. The mortality and survival groups showed overlapping scores (PSI: 151.50 vs. 137.00, p = 0.062; CURB-65: 3 vs. 2, p = 0.103), consistent with previous findings highlighting their diminished discriminatory power in older populations [22, 23]. The overemphasis on age and comorbidities in these scoring systems likely contributes to their reduced efficacy in stratifying risk among elderly patients, many of whom inherently cluster in higher-risk categories.

### Biological basis of IL-6 dynamics

IL-6's rapid response to pathophysiological stress and short half-life make it a sensitive biomarker for monitoring disease progression and therapeutic response [24–28]. Among inflammatory biomarkers, serum IL-6 shows the most rapid response to pathophysiological stress, with a relatively short half-life of approximately 15 hours [25–27]. In contrast, PCT responds more slowly with a longer half-life (approximately 24 h), while CRP increases even more gradually and peaks much later (after 20–72 h), with a half-life of approximately 19 hours [28–30].

This biological difference may explain why IL-6 dynamics showed significant discriminatory power within the first 48 h, while CRP and PCT changes did not distinguish between survivors and non-survivors as effectively. Despite the greater clinical availability of CRP and PCT testing in many settings, our findings suggest that the rapid and sensitive nature of IL-6 dynamics provides unique prognostic information that may not be captured by these traditional biomarkers in the early course of elderly CAP patients As demonstrated in Table 4, increased IL-6 levels within 48 h were associated with a substantially higher mortality rate (36.00% vs 8.93% in patients with decreased levels; OR = 5.39, p = 0.004). This strong association highlighted how IL-6's rapid response characteristics may allow for earlier identification of patients at high risk of deterioration, potentially providing a critical window for therapeutic intervention before clinical decline becomes evident through other parameters. Our findings align with previous studies demonstrating the association between persistent IL-6 elevation and poor outcomes in CAP patients [9, 19, 31, 32]. Our findings on early IL-6 dynamics within 48 h contribute to the understanding of inflammatory responses in elderly CAP patients.

### Optimized current scoring systems with IL-6 dynamics

To further illustrate the prognostic utility of IL-6 dynamics, we analyzed the data using both risk stratification and ROC curve analysis. Subgroup analysis revealed that IL-6 dynamics provided significant discriminatory power within high-risk categories. For instance, in the very high-risk PSI group (≥ 130), patients with increasing IL-6 levels demonstrated a mortality rate of 46.67%, compared to 12.00% in those with decreasing levels (OR = 22.06, p = 0.005). Similarly, within the CURB-65 moderate-risk group (score = 2), the mortality rate was 41.67% for patients with increasing IL-6 levels versus 9.52% for those with decreasing levels (OR = 21.27, p = 0.008). These findings suggested that IL-6 dynamics refine risk stratification even among patients already identified as high risk.

In addition to subgroup analysis, ROC curve evaluation demonstrated the added prognostic value of incorporating IL-6 dynamics into traditional severity scoring systems. IL-6 dynamics alone achieved an AUC of 0.7020 (p = 0.0179), outperforming PSI (AUC = 0.6631, p = 0.056) and CURB-65 (AUC = 0.6231, p = 0.1491). When combined with PSI, IL-6 dynamics improved the AUC to 0.7676 (p = 0.0017), and with CURB-65, the AUC increased to 0.7564 (p = 0.0027). These results demonstrate the potential of IL-6 dynamics to optimize existing clinical scoring systems, enhancing their accuracy in predicting mortality in elderly CAP patients.

By integrating both subgroup analysis and ROC curve assessment, our findings highlight the dual utility of IL-6 dynamics in providing actionable insights for clinical decision-making and robust statistical validation of prognostic models.

### Limitations and future directions

Several limitations should be noted. First, the small sample size (n = 81) may have limited the statistical power to detect subtle effects among other cytokines. Our post-hoc power analysis revealed that while our sample size provided adequate power (> 88%) to detect the predictive performance of individual combined models compared to the null hypothesis [33], it offered limited power to detect differences between correlated ROC curves (31.9–50.5%) [34]. This reflects inherent challenges in demonstrating incremental value in prediction models, particularly with small sample sizes. Detailed power analysis results are available in the Supplementary Information (Table S1).

Clinically relevant differences were observed in key parameters, including ICU admission rates and the need for ventilatory support, consistent with anticipated patterns of disease severity in elderly CAP patients. In this exploratory study, we emphasized effect sizes and clinical relevance alongside statistical significance, recognizing that overreliance on p-values can obscure important clinical patterns in heterogeneous elderly populations, particularly in preliminary studies with sample size constraints.

Furthermore, while our focus on IL-6 dynamics was supported by our data, we acknowledge limitations in comparing against more widely available biomarkers. CRP dynamics did not significantly enhance the predictive accuracy of traditional severity scores. Interpretation of the PCT analysis, however, was constrained by incomplete data availability (only available for 56.8% of patients).While the PSI + PCT model showed statistical significance in this subset of patients, the limited sample size, absence of significant dynamic changes in PCT between survival groups (Table 3), and incomplete data coverage constrain the reliability and generalizability of this finding. Future studies should systematically compare the prognostic value of IL-6 against these more accessible biomarkers with complete data collection, considering both clinical performance and cost-effectiveness.

Second, this was a single-center study, potentially limiting the generalizability of the findings. Third, as an observational study, causality between IL-6 dynamics and mortality cannot be inferred. Lastly, other cytokines or multi-biomarker approaches might offer additional prognostic value beyond IL-6 alone.

Future studies should involve larger, multicenter cohorts to validate these findings and explore the integration of IL-6 dynamics with other biomarkers. Such studies would provide greater statistical power and enable more robust comparative analyses between different biomarkers, particularly CRP and PCT. Comprehensive analyses of cytokine networks and their interplay with traditional biomarkers may further elucidate the underlying immunological mechanisms in elderly CAP patients and determine the most clinically practical and cost-effective risk stratification approaches. 

## Conclusion

The dynamic changes in interleukin-6 (IL-6) levels within the first 48 h of hospitalization show considerable potential as a prognostic marker for mortality in elderly patients with community-acquired pneumonia (CAP). In this pilot study, incorporating IL-6 dynamics into established severity scores, such as PSI and CURB-65, appeared to enhance prognostic accuracy and risk stratification for this vulnerable population. Although individual models showed promising predictive ability, our sample size limited the statistical power to detect inter-model differences. These findings underscored the critical role of dynamic biomarker monitoring in clinical practice, which may enable enabling more precise decision-making and potentially better patient outcomes. Validation through larger, multi-center studies is essential not only to confirm these results but also to robustly establish the incremental value of IL-6 dynamics over traditional severity scores and facilitate their integration into routine care. 

## Supplementary Information

Below is the link to the electronic supplementary material.

Supplementary file1 (DOCX 40 KB)

## Data Availability

The datasets generated and/or analyzed during the current study are available from the corresponding author on reasonable request.

## Abbreviations

CAP: Community-acquired pneumonia
IL-6: Interleukin-6
PSI: Pneumonia severity index
CURB-65: Confusion, urea, respiratory rate, blood pressure, age ≥ 65
AUC: Area under the curve
ICU: Intensive care unit
CRP: C-reactive protein
WBC: White blood cell
TNF-α: Tumor necrosis factor alpha
IFN-γ: Interferon gamma
GM-CSF: Granulocyte–macrophage colony-stimulating factor
MCP-1: Monocyte chemoattractant protein-1
MIP-1α: Macrophage inflammatory protein-1 alpha
