# Correction: Dynamic cytokine monitoring enhances CAP severity scores in elderly patients: a prospective pilot study

**DOI:** 10.1007/s11739-025-04053-8

**Published:** 2025-07-21

**Authors:** Cheng-Han Chen, Yi-Tzu Lee, Ching-Fen Shen, Chao-Min Cheng

**Affiliations:** 1 Department of Emergency Medicine, Taipei Veterans General Hospital, Taipei, Taiwan; 2 School of Medicine, National Yang Ming Chiao Tung University, Taipei, Taiwan; 3 Department of Pediatrics, National Cheng Kung University Hospital, College of Medicine, National Cheng Kung University, Tainan, Taiwan; 4 Institute of Clinical Medicine, College of Medicine, National Cheng Kung University, Tainan, Taiwan; 5 Institute of Biomedical Engineering, National Tsing Hua University, Hsinchu, Taiwan

**Correction: Internal and Emergency Medicine** 10.1007/s11739-025-03975-7

The text under the method-Cytokine analysis section for this article was incorrectly given as ‘Bio-Plex Mouse Cytokine Group I, 23-plex assay kit’ but should have been ‘Bio-Plex Pro Human Cytokine 27-plex Assay (Bio-Rad Laboratories, Hercules, CA, #M500KCAF0Y)’.

The original article has been corrected.

