# Supplementary material for: Dynamic cytokine monitoring enhances CAP severity scores in elderly patients: a prospective pilot study
Source: Intern Emerg Med. 2025 May 27;21(2):653–64. doi: 10.1007/s11739-025-03975-7 (PMC13061778; doi:10.1007/s11739-025-03975-7)
Supplement: Supplementary file 1 — Supplementary file1 (DOCX 40 KB) [file 11739_2025_3975_MOESM1_ESM.docx]

Dynamic Cytokine Monitoring Enhances CAP Severity Scores in Elderly Patients: A Prospective Pilot Study

Supplementary Information

Cheng-Han Chen^1,2*^, Yi-Tzu Lee^1,2^, Ching-Fen Shen^3,4^, Chao-Min Cheng^5*^

^1^Department of Emergency Medicine, Taipei Veterans General Hospital, Taipei, Taiwan

^2^School of Medicine, National Yang Ming Chiao Tung University, Taipei, Taiwan

^3^Department of Pediatrics, National Cheng Kung University Hospital, College of Medicine, National Cheng Kung University, Tainan, Taiwan

^4^Institute of Clinical Medicine, College of Medicine, National Cheng Kung University, Tainan, Taiwan

^5^Institute of Biomedical Engineering, National Tsing Hua University, Hsinchu, Taiwan

***Table S1: Post-hoc Power Analysis for ROC Curve Models***

| **A. Power Analysis for Individual Predictive Models** | | | | | |  |
| --- | --- | --- | --- | --- | --- | --- |
| **Predictive Model** | | AUC | Sample Size (n=81) | α Level | Power (%) | Reference Hypothesis (AUC) |
| **PSI** | | 0.6631 | 14 events, 67 non-events | 0.05 | 50.5 | 0.5 |
| **CURB-65** | | 0.6231 | 14 events, 67 non-events | 0.05 | 31.9 | 0.5 |
| **IL-6 Change** | | 0.7020 | 14 events, 67 non-events | 0.05 | 68.7 | 0.5 |
| **PSI + IL-6 Change** | | 0.7676 | 14 events, 67 non-events | 0.05 | 90.8 | 0.5 |
| **CURB-65 + IL-6 Change** | | 0.7564 | 14 events, 67 non-events | 0.05 | 88.0 | 0.5 |

| **B. Power Analysis for Comparisons Between Correlated ROC Curves** | | | | | |  |
| --- | --- | --- | --- | --- | --- | --- |
| **Comparison** | | AUC Difference | Sample Size (n=81) | α Level | Power (%) | Method |
| **PSI vs. PSI + IL-6 Change** | | 0.1045 | 14 events, 67 non-events | 0.05 | 50.5 | DeLong's test |
| **CURB-65 vs. CURB-65 + IL-6 Change** | | 0.1333 | 14 events, 67 non-events | 0.05 | 31.9 | DeLong's test |

| **C. Sample Size Requirements for ROC Curve Analysis** | | | |  |
| --- | --- | --- | --- | --- |
| **Analysis Type** | | Minimum Recommended Events | Study Events | Adequacy Assessment |
| **Preliminary ROC analysis** | | 10-15 events | 14 events | Meets minimum requirement |
| **Robust ROC analysis** | | >30 events | 14 events | Below recommended threshold |
| **Model comparison** | | >50 events | 14 events | Substantially below recommended threshold |

*Note*

*1. Power analysis was conducted using methods described by Zhou et al. (2011) in "Statistical Methods in Diagnostic Medicine."^1^*

*2. For individual predictive models (Section A), power was calculated for detecting an AUC significantly different from 0.5 (random classification)^2^.*

*3. For comparisons between correlated ROC curves (Section B), power was calculated using DeLong's method for the observed differences between models^3^.*

*4. The substantially lower power for model comparisons reflects the statistical challenges in detecting incremental value in prediction models, particularly with small sample sizes and when models share common components.*

*5. While our sample size meets the minimum threshold for preliminary ROC analysis, the results should be interpreted as hypothesis-generating and require validation in larger cohorts^4^.*

| **Pathogen** | **Survivors (n=67)** | **Non-survivors (n=14)** | **Total (n=81)** |
| --- | --- | --- | --- |
| **Pseudomonas species** | 2 (3.0%) | 4 (28.6%) | 6 (7.4%) |
| **Staphylococcus species** | 2 (3.0%) | 3 (21.4%) | 5 (6.2%) |
| **Chlamydophila species** | 2 (3.0%) | 2 (14.3%) | 4 (4.9%) |
| **Streptococcus species** | 4 (6.0%) | 1 (7.1%) | 5 (6.2%) |
| **Klebsiella species** | 6 (9.0%) | 1 (7.1%) | 7 (8.6%) |
| **Haemophilus species** | 1 (1.5%) | 0 (0.0%) | 1 (1.2%) |
| **Mycoplasma species** | 2 (3.0%) | 0 (0.0%) | 2 (2.5%) |
| **Other bacteria** | 1 (1.5%) | 0 (0.0%) | 1 (1.2%) |
| **Identified pathogens** | 20 (29.9%) | 11 (78.6%) | 31 (38.3%) |
| **No pathogen identified** | 47 (70.1%) | 3 (21.4%) | 50 (61.7%) |

***Table S2: Microbial Etiology of Community-Acquired Pneumonia by Survival Status***

*Note: Percentages are calculated within each column. The significantly higher rate of pathogen identification in non-survivors (78.6% vs. 29.9%, p<0.001) may reflect greater bacterial burden or more severe infection.*

***Table S3: Initial Antibiotic Treatment by Survival Status***

| **Antibiotic Class** | **Survivors (n=67)** | **Non-survivors (n=14)** | **Total (n=81)** |
| --- | --- | --- | --- |
| **Cephalosporins** | 31 (46.3%) | 3 (21.4%) | 34 (42.0%) |
| **Penicillin** | 20 (29.9%) | 3 (21.4%) | 23 (28.4%) |
| **Quinolones** | 11 (16.4%) | 3 (21.4%) | 14 (17.3%) |
| **Carbapenems** | 5 (7.5%) | 5 (35.7%) | 10 (12.3%) |

*Note: All patients received antibiotic treatment according to IDSA guidelines for CAP. The higher proportion of carbapenem use in non-survivors likely reflects more severe presentations or concerns about resistant pathogens.*

***Table S4: Comparison of Predictive Models Incorporating Traditional Inflammatory Markers***

| **Model** | **AUC** | **SE** | **95% CI (Lower)** | **95% CI (Upper)** | **p value** |
| --- | --- | --- | --- | --- | --- |
| **CURB-65 & CRP Change** | *0.6152* | *0.08238* | *0.4537* | *0.7767* | *0.2172* |
| **CURB-65 & PCT Change^†^** | *0.6740* | *0.09435* | *0.4891* | *0.8589* | *0.1463* |
| **CURB-65 & IL-6 Change** | 0.7564 | 0.06509 | 0.6288 | 0.8840 | 0.0027^*^ |
| **PSI & CRP Change** | *0.6471* | *0.07522* | *0.4996* | *0.7945* | *0.1152* |
| **PSI & PCT Change^†^** | *0.7527* | *0.08278* | *0.5905* | *0.9150* | *0.0348** |
| **PSI & IL-6 Change** | 0.7676 | 0.06889 | 0.6326 | 0.9026 | 0.0017^*^ |

**p < 0.05 ^†^PCT data available for 46 patients only (56.8% of the cohort)*

*Note: This table compares the predictive performance of models combining traditional severity scores with commonly available inflammatory markers. While PSI & PCT Change showed statistical significance (p=0.0348), this analysis was limited by incomplete PCT data availability. These models generally demonstrated lower discriminatory power than the IL-6-based models.*

***Reference***

1. Zhou X-h, Obuchowski NA, McClish DK. *Statistical methods in diagnostic medicine*. 2nd ed. Wiley series in probability and statistics. Wiley; 2011.

2. Hanley JA, McNeil BJ. The meaning and use of the area under a receiver operating characteristic (ROC) curve. *Radiology*. Apr 1982;143(1):29-36. doi:10.1148/radiology.143.1.7063747

3. DeLong ER, DeLong DM, Clarke-Pearson DL. Comparing the areas under two or more correlated receiver operating characteristic curves: a nonparametric approach. *Biometrics*. Sep 1988;44(3):837-45.

4. Peduzzi P, Concato J, Kemper E, Holford TR, Feinstein AR. A simulation study of the number of events per variable in logistic regression analysis. *J Clin Epidemiol*. Dec 1996;49(12):1373-9. doi:10.1016/s0895-4356(96)00236-3
